# Personal

**Published:** 1910-07

**Authors:** 


### PERSONAL.

---

Dr. Adam H. Wright, of Toronto, president of the Canadian Medical Association at its recent session held in Toronto, chose for the subject of his presidential address, "The General Practitioner." Professor Wright made favorable reference in his address to the paper by Dr. Mann, of Buffalo, on "Dividing Professional Fees," read before the Medical Society of the County of Erie last February, and published in the issue of the Journal for April, 1910.

---

Dr. and Mrs. Julius H. Potter, of Dearborn Street, Buffalo, with a party of New York friends, start the first of July to spend several months in European travel.

Dr. John Benjamin Murphy, of Chicago, was elected president of the American Medical Association at its recent meeting in Saint Louis, a fitting recognition of the eminent surgeon's distinguished ability. Should the association meet in Buffalo in 1912, Dr. Murphy will preside during the first day's session of the house of delegates, and during the initial proceedings at the open session on Tuesday.

Dr. Henry Schwarz, of Saint Louis, delivered the address of welcome on behalf of the Saint Louis Medical Society to the American Medical Association at its recent annual meeting. In phraseology, in material, in good form and in length, the address of Dr. Schwarz has rarely been equaled by any similar address on a like occasion, and it may well serve as a model for the future.

Dr. George W. Crile, of Cleveland, was elected to the Chairmanship of the Section on Surgery at the recent meeting of the American Medical Association in Saint Louis.

Dr. Edgar A. Vander Veer, of Albany, has been appointed a member of the medical board of the Champlain Valley Hospital, vice Dr. Irving S. Haynes, of New York.

